# Supplementary figures and images for: Transcription factor interactions explain the context-dependent activity of CRX binding sites
Source: PLoS Comput Biol. 2024 Jan 16;20(1):e1011802. doi: 10.1371/journal.pcbi.1011802 (PMC10817189; doi:10.1371/journal.pcbi.1011802)

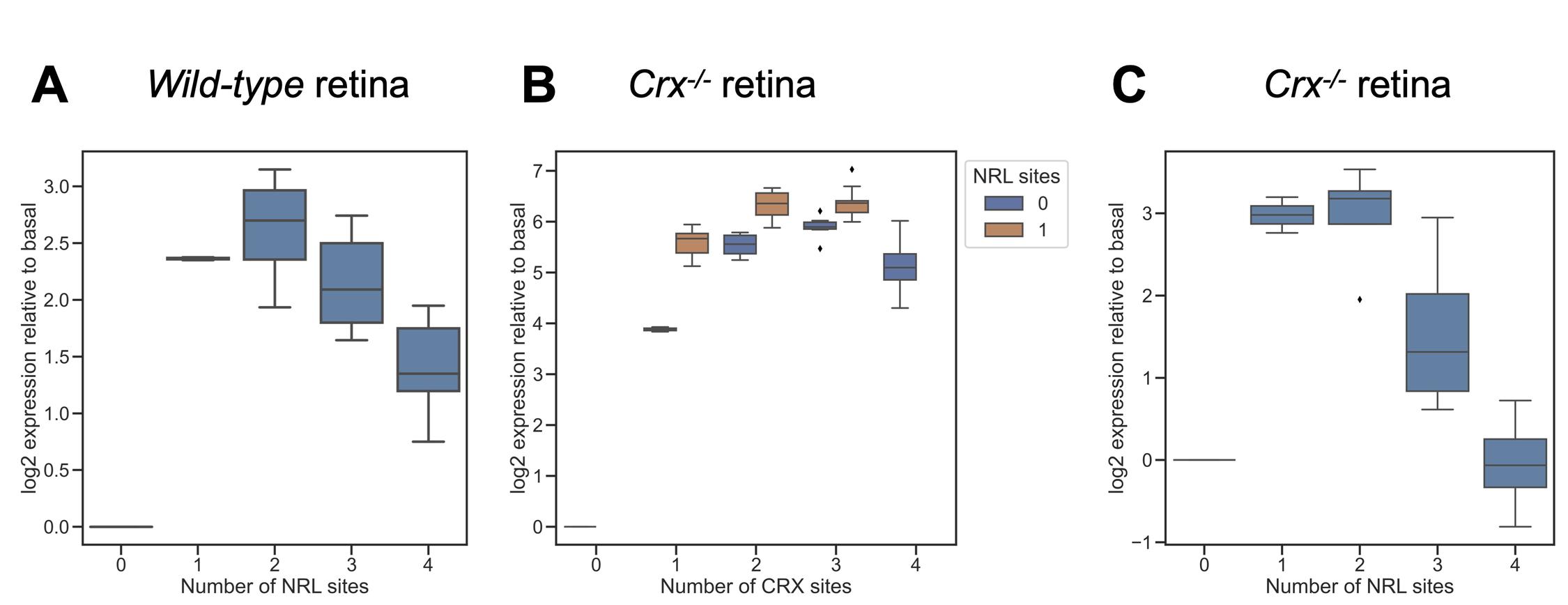

Supplement: S1 Fig — Synthetic CREs composed only of NRL sites show an increase, then a decrease in activity relative to the Rho basal promoter as the number of sites is increased. Plot shows a subset of the data reported in [13]. (TIF) [file pcbi.1011802.s001.tif]

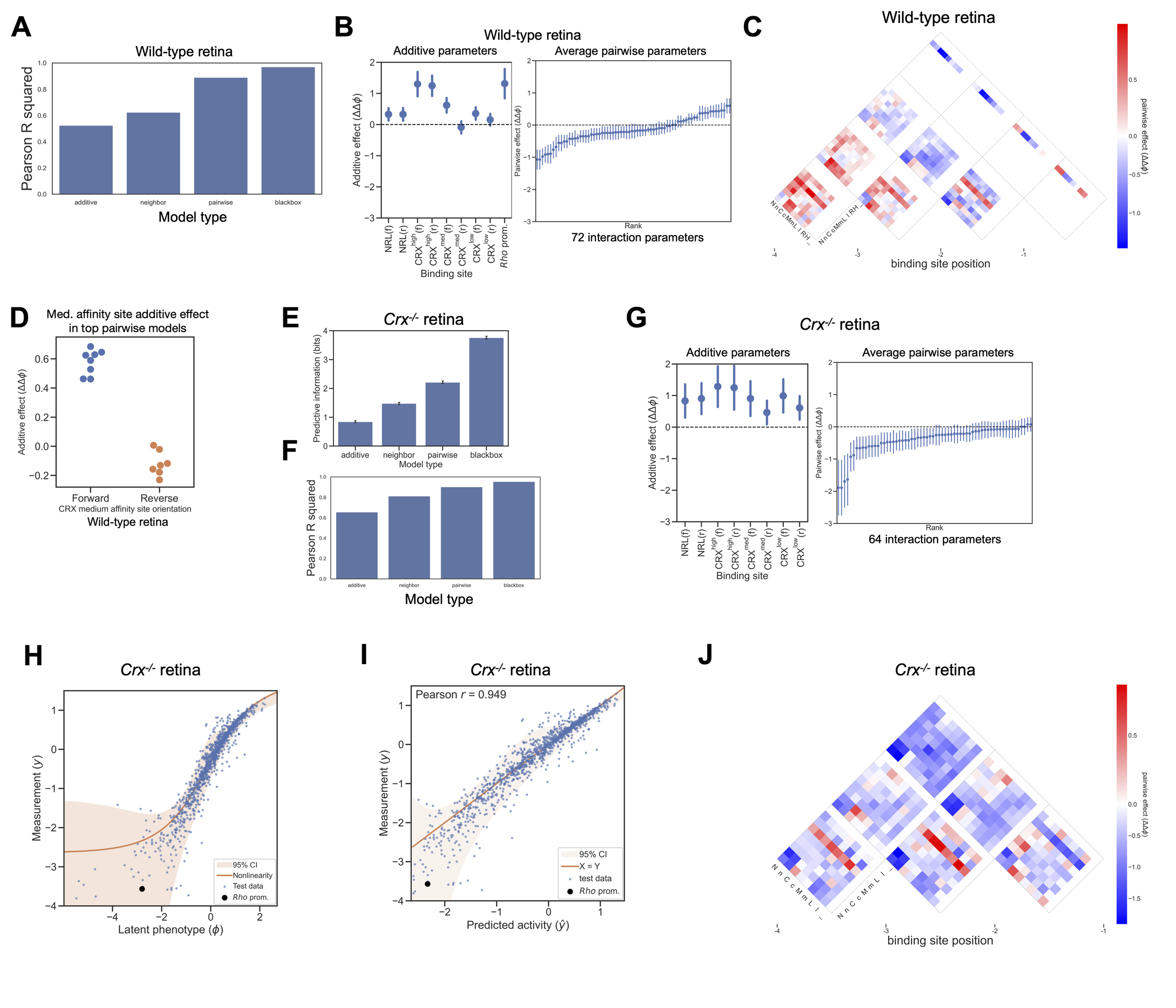

Supplement: S2 Fig — (A) The performance of different model architectures measured by Pearson correlation coefficients, wild-type retina. (B) Estimates of model parameter uncertainties for additive (left) and pairwise interaction (right) parameters, generated by the built-in MAVE-NN function to estimate uncertainties. Interaction parameters are ordered by rank for visualization. (C) Model parameters for position-specific pairwise contributions of CRX and NRL sites in wild-type retina. Forward and reverse orientation of binding sites is indicated by capital or lower case letter. CRX sites are either high (C or c), medium (M or m), or low (L or l) affinity. NRL sites are labeled N or n and the Rho promoter is labeled R. There are no model parameters for Hsp68 (H, used as the overall basal sequence) or the placeholder site _ used to equalize the lengths of input sequences. See methods for details. (D) Anomalous activity of reverse medium affinity CRX site, compared to the forward site. Additive parameters shown for independently trained pairwise models initialized from different random seeds (n = 8). (E, F) Performance of different model architectures fit to MPRA measurements of the CRX-NRL library in Crx-/- retina. (G) Estimates of model parameter uncertainties for additive (left) and pairwise interaction (right) parameters in Crx-/- retina. (H) Observed activity (y-axis) of test set sequences vs the latent phenotype inferred by the pairwise model of Crx-/- retina. (I) Observed activity (y-axis) of test set sequences vs predicted activity of the pairwise model of Crx-/- retina. (J) Position-specific pairwise contributions of CRX and NRL sites to activity in Crx-/- retina. (TIF) [file pcbi.1011802.s002.tif]

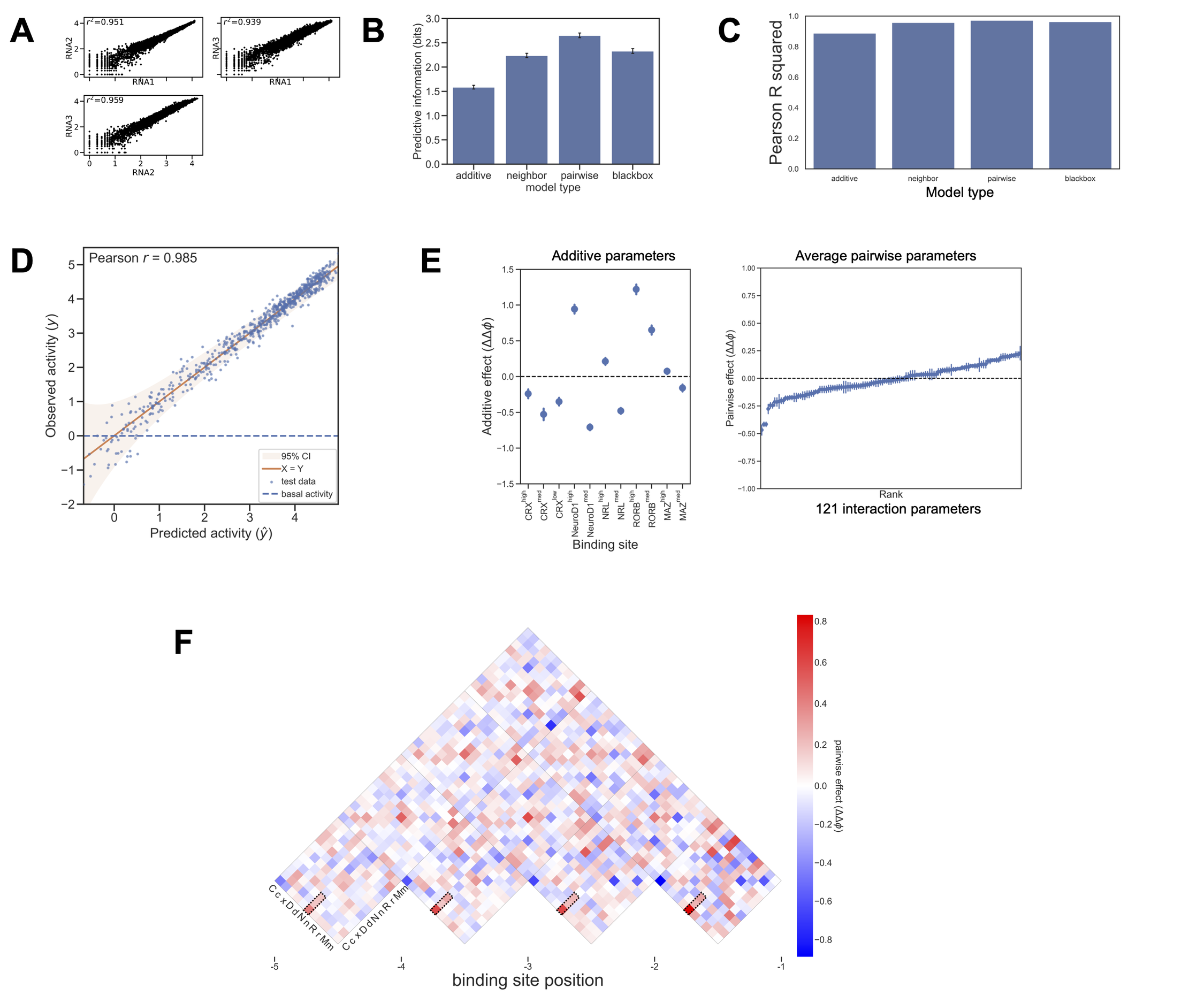

Supplement: S3 Fig — (A) Reproducibility of MPRA measurements across three replicates. (B,C) Performance of modes fit to measurements of the MPRA library of CREs composed of five TFBSs, expressed in terms of predictive information and Pearson correlation. (D) Observed activity (y-axis) of test set sequences compared to the activity predicted by the pairwise model (x-axis). (E) Estimates of model parameter uncertainties for additive (left) and pairwise interaction (right) parameters. Interaction parameters are ordered by rank for visualization. (F) Position-specific pairwise contributions of diverse TF binding sites. Capital and lowercase letters represent high and medium affinity sites for CRX (C), NEUROD1 (D), NRL (N), RORB (R), and MAZ (M). Low affinity CRX sites are represented by x. Dashed boxes indicate strong cooperative interactions of high affinity NRL sites with CRX sites. (TIF) [file pcbi.1011802.s003.tif]

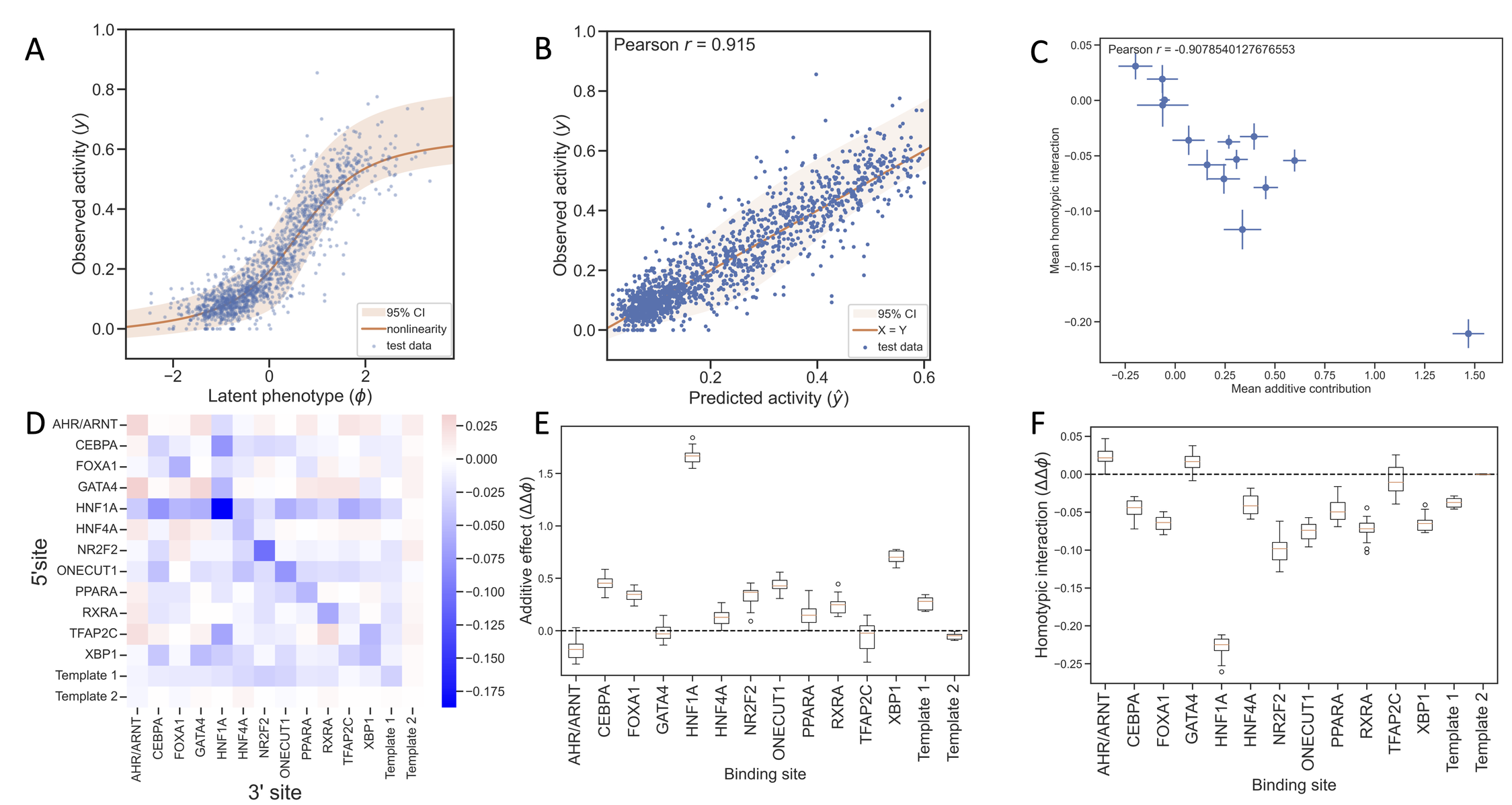

Supplement: S4 Fig — (A) Observed activity (y-axis) of test set sequences compared to the latent phenotype (x-axis) predicted by the pairwise model. (B) Observed activity (y-axis) of test set sequences compared to the activity predicted by the pairwise model (x-axis). (C) Mean homotypic interaction contribution of each TF plotted against mean additive contribution. Error bars are standard deviations across 20 simulated replicates generated by MAVE-NN’s built-in parameter uncertainty estimation function. (D) Model parameters representing pairwise contributions of TFBSs averaged across positions. (E) Distributions over simulated replicates of mean additive contribution from each TF averaged across positions. (F) Distributions over simulated replicates of mean homotypic interaction contribution from each TF averaged across pairs of positions. (TIF) [file pcbi.1011802.s004.tif]

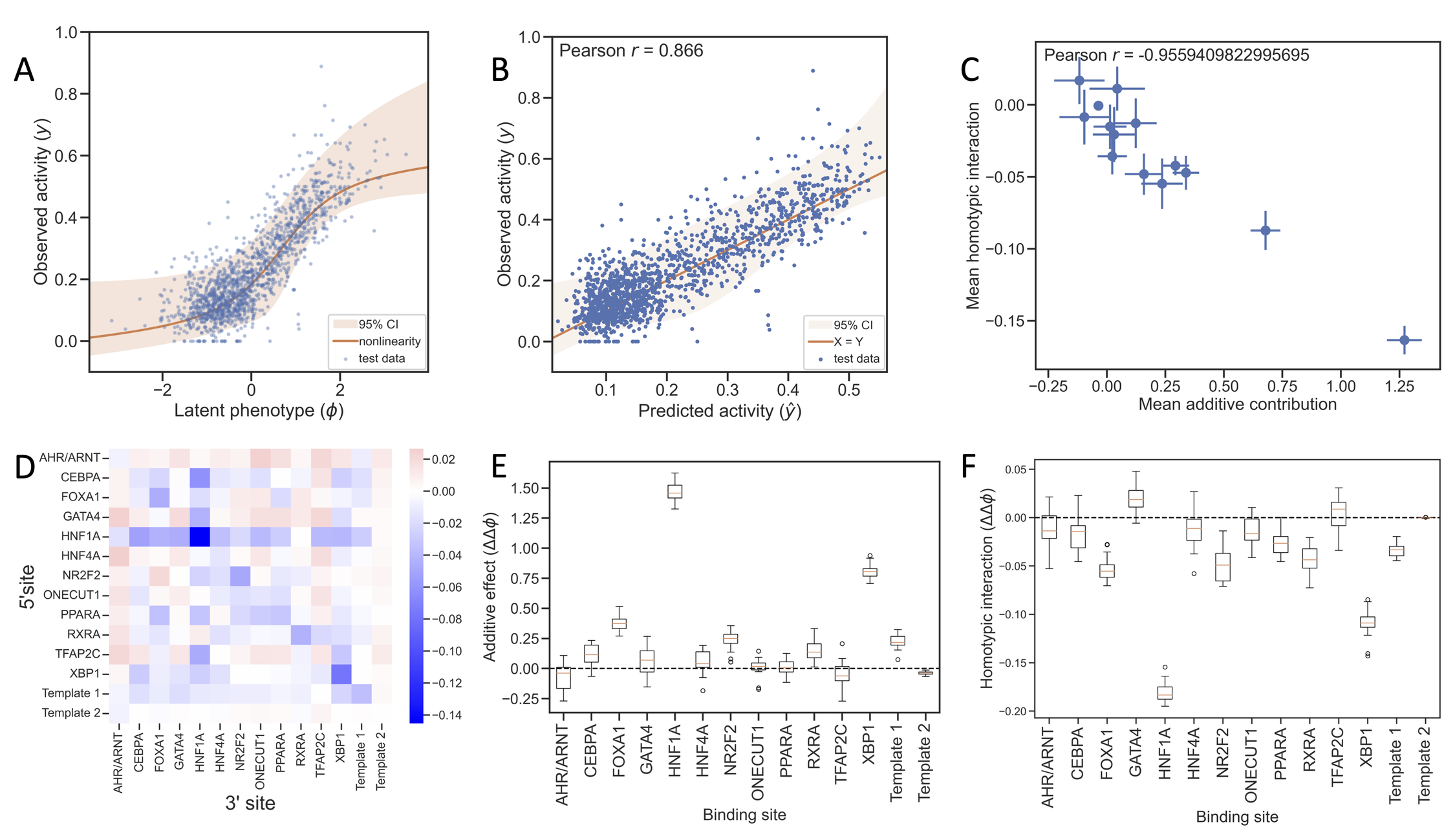

Supplement: S5 Fig — (A) Observed activity (y-axis) of test set sequences compared to the latent phenotype (x-axis) predicted by the pairwise model. (B) Observed activity (y-axis) of test set sequences compared to the activity predicted by the pairwise model (x-axis). (C) Mean homotypic interaction contribution of each TF plotted against mean additive contribution. Error bars are standard deviations across 20 simulated replicates. (D) Model parameters representing pairwise contributions of TFBSs averaged across positions. (E) Distributions over simulated replicates of mean additive contribution from each TF averaged across positions. (F) Distributions over simulated replicates of mean homotypic interaction contribution from each TF averaged across pairs of positions. (TIF) [file pcbi.1011802.s005.tif]

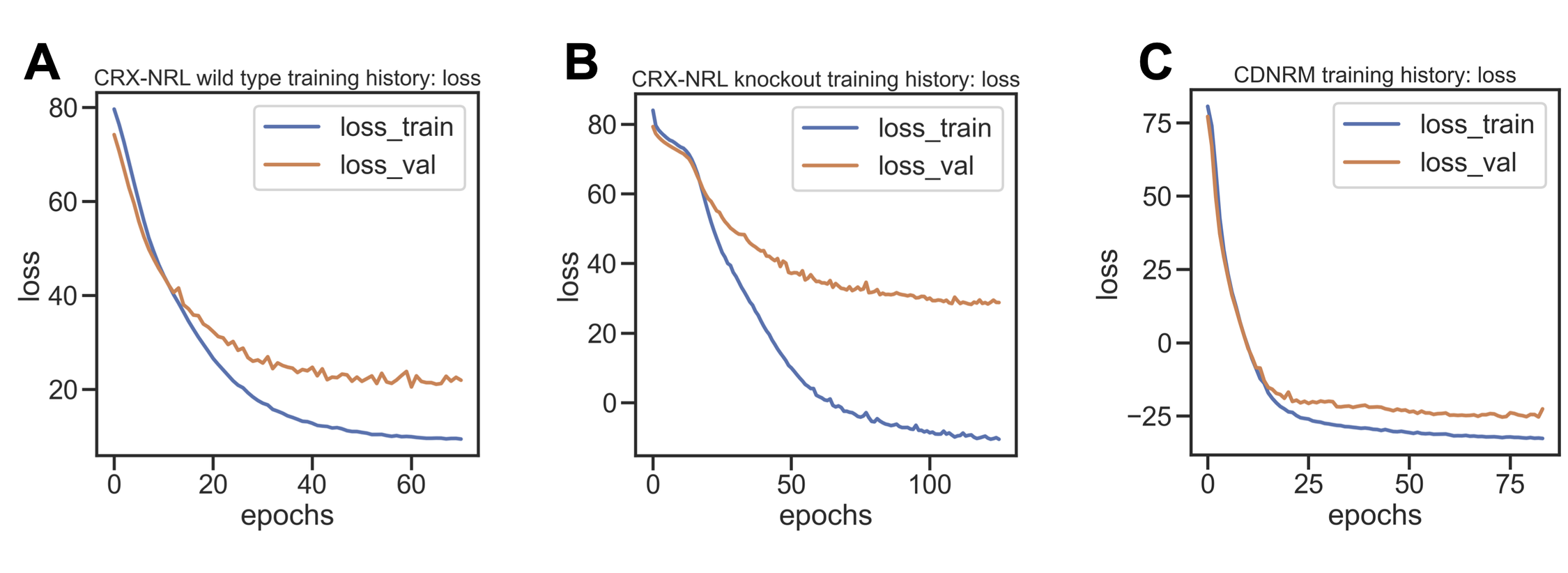

Supplement: S6 Fig — Training and validation set loss by epoch for MAVE-NN pairwise interaction models for (A) CRX-NRL library in wild type retina, (B) CRX-NRL library in Crx-/- retina, and (C) CDNRM library in wild-type retina. (TIF) [file pcbi.1011802.s006.tif]
