# Supplementary material for: Transcription factor interactions explain the context-dependent activity of CRX binding sites
Source: PLoS Comput Biol. 2024 Jan 16;20(1):e1011802. doi: 10.1371/journal.pcbi.1011802 (PMC10817189; doi:10.1371/journal.pcbi.1011802)
Supplement: Attachment — Submitted filename: Comments Loell et al.pdf [file pcbi.1011802.s007.pdf]

Review of Loell et al.

The authors have investigated the context-dependent activity of transcription factor (TF) CRX that contribute to gene activation or repression. For this, the authors have utilized massively parallel reporter assays (MPRAs) to measure the activity using synthetic cis-regulatory elements and then analyzed the measurements using MAVE-NN, neural network-based models. This work is a good effort in understanding the role of DNA sequence and its context in gene regulation and the authors aim to perform this in a systematic manner using synthetic regulatory elements in MPRAs. The authors report that for gene activation, positive heterotypic interactions involving different TFs are important and allows to overcome the negative effects from the homotypic interactions of the same TF in this study. This is a good analysis, allowing easy interpretation of their results to a general audience and also for those working in the field of gene regulation. I have few major concerns that should be addressed to make the findings consistent and robust.

1. The authors have modeled their MPRA measurements and make their interpretation using only one neural-network based model *i.e.* MAVE-NN. Although the authors have listed their rational behind using MAVE-NN, there are already many different published tools to model and analyze MPRA data. Thus, it is important that the authors compare their measurements and results using few other tools for example, DEEP-STARR (PMID: 35551305) with details of the hyperparameters used to fit the models.

2. One of the major limitations and what the authors have also noted in their manuscript is the lack of MPRA measurements without the basal *Rho* promoter which has multiple CRX sites. It would be best to perform this assay using a library without the *Rho* promoter and then model the effects both for the positive and negative interactions. This is not a big experiment and should have been done as new MPRA designs with synthetic CREs comprising of multiple different TFs were done for the results shown in Fig. 3

3. It was also not clear which experiments and to what extent the MPRA data using *Hsp68* promoter data was used. Comparison of results from both these results will also allow the authors to dissect the effects of the basal promoter used in their study. What is the correlation of MPRA activity from the two basal promoters i.e. *Rho* and *Hsp68* and are the results consistent for example with the measured activity in terms of number of CRX or NRL sites?

4. How do the authors explain their results in terms of biological context? Lot of different lineage-specific TF motifs were included in the synthetic CREs and measured but it would be good to know their function *in vivo*. For example, lot of these effects can be analyzed and measured in retinal pigmented epithelial (RPE) cell line. One way would be to look at the publicly available RPE cell line data such as ATAC-seq or if appropriate ChIP-seq data is available and can correlate the *cis*-regulatory grammar around the active and inactive genes. If such a grammar exists, then other way would be to use CRISPR to measure the effects CREs on gene activation or repression. This for sure would greatly improve the strength and outreach of this work.
