# Supplementary material for: Transcription factor interactions explain the context-dependent activity of CRX binding sites
Source: PLoS Comput Biol. 2024 Jan 16;20(1):e1011802. doi: 10.1371/journal.pcbi.1011802 (PMC10817189; doi:10.1371/journal.pcbi.1011802)
Supplement: Attachment — Submitted filename: Loell.Response_to_reviews.pdf [file pcbi.1011802.s009.pdf]

Dear Dr. Liu,

We are submitting our revised manuscript “Transcription factor interactions explain the context-dependent activity of CRX binding sites.” We revised the manuscript to address the remaining minor comments of the reviewers. We discuss our changes below, and we have attached a marked-up copy of the manuscript showing all changes.

<u>**Response to reviews**</u>

Reviewer #2: I am satisfied with the authors response to my comments and can recommend this for acceptance.

Reviewer #3: I commend the authors for thoughtful responses to the critiques, additional analyses and improved communication of the work. The manuscript is much strengthened by the revision.

Minor concerns:
• Fig. 3D – the x-axis labels are shifted relative to the bar plots.

<u>Response:</u> We fixed the x-axis labels in Fig. 3D and have uploaded the revised figure.

• For the HepG2 and liver MPRA datasets, “pooled data” means that the RNA and DNA counts were summed as opposed to averaged across replicates? Please clarify. (Analysis of these additional contexts greatly strengthened the work.)

<u>Response:</u> We clarified our description of the procedure. Rather than averaging across replicates, we treated both replicates as part of one pooled dataset that we randomly divided into training, validation and test sets. As a result, there were multiple independent measurements per CRE in the dataset.

See lines 430-433 in the revised text.

• Authors describe parameter uncertainty estimates in figure legends (e.g., Fig. S4C) as “standard parametric bootstrap replicates”. However, based on reading the methods (model fitting paragraph in Methods), “bootstrapping” (subsampling the underlying data with replacement to generate a distribution of parameters) was not performed. The methodology for modeling parameter uncertainty does not subsample the underlying MPRA data and therefore does not account for variability / noisy datapoints in that data. Parameter uncertainty needs to be achieved by subsampling / bootstrapping the underlying data and fitting multiple models and assessing parameter distributions across subsamples. Here, variability in parameters is estimated by fitting data simulated by a single MAVE-NN model fit to all data, so the parameter uncertainty is underestimated by this method, as (1) all data was used to fit the MAVE-NN model used for simulation (so no assessment of biological or technical variability of data) and (2) the MAVE-NN model is a simplified representation of the biology and therefore simulated datasets are unlikely to be representative of biological datasets. This impacts figures showing variability in parameters, where parameter uncertainties are likely

underestimates. In terms of addressing this issue, I don't think the uncertainty estimates need to be re-evaluated, just clarify what was done and limitations. Authors could simply not using the word bootstrap (if bootstrap was not performed) in results / figure legends and instead pointing readers to methods. They might also add a sentence about caveats of their uncertainty measurements (underlying MPRA data was not subsampled, likely lower bound on uncertainty), so readers are informed.

Response: We understand the reviewer's concern and have eliminated the word 'bootstrap' in all descriptions in the main text and figure legends, except where necessary to refer to the name of MAVE-NN's built-in 'bootstrap' function. We have revised the methods section to clarify the procedure used by MAVE-NN to estimate parameter uncertainties and we have noted that the estimates are likely a lower bound.

In the revised Methods, we briefly summarize the justification of the MAVE-NN authors for their approach to estimating parameter uncertainties. In the original paper (Tareen, et al. 2022, PMID: 35428271), the authors give several reasons for the approach. The most relevant one to the current paper is as follows:

"There are good reasons to estimate parameter uncertainties using this parametric bootstrap approach, rather than through other bootstrapping methods. In particular, the parametric bootstrap keeps the sequences in each simulated data set fixed, and treats only the measurements as being stochastic. This comports with the fact that, in many MAVE designs, a specific set of user-specified sequences are assayed, with each sequence receiving a single measurement value. If each simulated dataset were instead generated by resampling sequence-measurement pairs from the full dataset, virtually all of these simulated datasets would end up lacking measurements for a substantial subset of the original assayed sequences. Among other issues, this can lead to parameter non-identifiability in models trained on simulated data even when such non-identifiability is absent in models trained on the original dataset."

See lines 730-738 for the new methods section. The word 'bootstrap' was eliminated from the main text and from the supplemental figure captions.

Thoughts for consideration:

• Would be nice to also visualize the Crx-/- MPRA data as was done for Fig. 1B. This will provide useful intuition that complements model parameters in Fig. 2. It would also be compelling to visualize the data this way for the other MPRA datasets modeled in this study, as these would support the "simple model" represented in Fig. 4.

Response: We added similar plots for the Crx-/- data and placed them in Fig S1B and C. The MPRA data supports the differences in parameters of the Crx-/- model. See lines 322-324 for the revised text.

For the library with four additional retinal TFBSs, we plot MPRA activities in Fig. 3E and show that these activities are consistent with the heterotypic and homotypic interactions inferred by the model. For example, Fig. 3E shows that CREs with only two CRX sites are more active on average than CREs with three CRX sites, a result that is consistent with the simple model of

Fig. 4. However, because the number of TFBSs is not varied as systematically in this library as in the other MPRA libraries we analyze, we cannot plot the equivalent of Fig. 1B

For the MPRA dataset from Smith, et al. 2013 (PMID: 23892608), we have added reference to specific figures in the original paper that show patterns related to our Figure 1B. Figure 2 of that paper shows the effects of systematically increasing the number of binding sites in a homotypic cluster, while Figure 3 shows that heterotypic TFBS clusters drive stronger expression than homotypic clusters. Because these plots are in the original paper, we don't believe it would be appropriate to replot the data in the manuscript. See lines 446 and 450-453 for revisions.

• I like the idea of simple models from Fig. 4 being used to analyze, e.g., ATAC-seq data, predicting whether chromatin accessibility regions are likely to be enhancers or silencers based on counts of homotypic and heterotypic motif occurrences. Could be the focus of future work, but it would be nice to know qualitatively (perhaps using Spearman correlation) to quantify agreement of this simple model with the 5 MPRA datasets used in this study. If the simple models perform reasonably, this would be big for transcriptional regulatory network inference using ATAC-seq data.

<u>Response:</u>

We quantified the agreement of the simple model (eq. 1) with the MPRA datasets in several ways:

1. For the CRX-NRL library in wild type retina (presented in Figure 2), the Spearman correlation between predicted and measured activity is 0.76. In the Crx-/- retina, this correlation reduces to 0.18, due to the loss of the strongest homotypic interactions in this context.
2. For the library of liver TFBSs from reference 68 (Figures S4 and S5), the Spearman correlations are lower, 0.53 (Mouse assay) and 0.43 (HepG2 cell assay), presumably because the simple model doesn't capture effects of order, orientation, spacing, etc. in these longer more complex CREs.
3. We used the simple model to predict MPRA patterns among genomic CREs (taken from our prior dataset, Friedman et al 2021). We made a plot similar to Fig. 1B, showing activities of CREs with increasing numbers of CRX sites, with and without additional photoreceptor TFBS. We find that (1) genomic CREs recapitulate the non-monotonic, repressive pattern seen with synthetic CREs and (2) the simple model predicts this pattern. The results are in a new figure panel 4C.

In each case, we used the unweighted simple model in which the constants in eq. 1 are all set to 1. For the MPRA library discussed in Figure 3, the simple model only predicts two different activities corresponding to the 2-2-1 and 3-1-1 CRE types shown in Figure 3A. This illustrates a limitation of the simple model, which doesn't account for order, orientation, spacing, and more complex interactions.

For the revisions, see lines 513-526 and 566-574.

Reviewer #4: The revised manuscript shows significant improvement and the authors have done a commendable job in addressing the previous comments. However, before its acceptance, I would like to suggest a few minor modifications to further enhance the clarity and completeness of the manuscript:

1. The authors should provide additional clarification regarding the specificity of their results. Are all the results cell-specific? If yes, the authors should indicate it in the very beginning of the results.

Response: The models are cell type specific since they are trained on cell type specific MPRA data. But we argue that the broader trends we identify, particularly negative homotypic interactions, occur in multiple cellular contexts, since we observe the phenomenon in multiple cell types.

We revised the text at various places to clarify the cell context in which each MPRA dataset was generated. See the changes tracked in the following lines:

123-124, 139, 142, 422

2. The preprocessing procedures and assumptions of the MAVE-NN model need to be explicitly defined in the manuscript. This will ensure that the methodology is transparent and reproducible.

Response:

For MPRA data from previously published papers (White et al, 2016 and Smith et al 2013), we used data taken directly from the supplementary files of the respective papers, without further processing. We clarified this in the first sentence of the Methods section under Model fitting. For the MPRA data published here for the first time, we describe our MPRA data processing methods in Methods section under MPRA data processing. See lines 702-703 and 791-798.

We added a summary of the assumptions underlying MAVE-NN in the results section. See lines 177-194.

3. The rationale behind using MAVE-NN, currently located in the discussion section, would be more appropriately placed in the introduction. This would provide the necessary context before presenting the results of the MAVE-NN.

Response: We have a brief statement of the rationale for MAVE-NN in the introduction. See lines 109-122.

We added a more extensive rationale at the beginning of the Results section when we describe the assumptions of MAVE-NN (see response to comment #2 above). See lines 177-194.

4. Could the authors specify the sample size for each dataset used in the study including real ones? This information is crucial for understanding the robustness of the predicted results.

<u>Response:</u> We added the library size for each MPRA library to the Methods section. See lines 702-706.

5. Lastly, it appears that the contribution of CRX binding sites to the activation or repression of transcription is inferred from the sign of parameters. Are these conditional activations or repressions? I recommend that the authors discuss the limitations of such interpretations in the discussion.

We added a discussion of our interpretation of the sign of the parameters in the Discussion section 622-627. For the models trained on the CRX/NRL dataset (Figure 2), the weights of the model were calibrated to the basal promoter using the gauge hyperparameter of MAVE-NN. We are thus confident that the signs of the additive and interacting terms in these models do in fact reflect a distinction between activation and repression. For the other MPRA datasets, we did not have basal promoter measurements for calibration. But we argue that the consistency of the model parameters with those of the calibrated models support our interpretation of the parameter signs. The interpretation is also supported by the measured effects of sequentially adding sites to homotypic clusters, which we show in Figure 1B, Figure S1A-C, and Figure 4C.
