# Supplementary material for: Transcription factor interactions explain the context-dependent activity of CRX binding sites
Source: PLoS Comput Biol. 2024 Jan 16;20(1):e1011802. doi: 10.1371/journal.pcbi.1011802 (PMC10817189; doi:10.1371/journal.pcbi.1011802)
Supplement: Attachment — Submitted filename: Loell.review_response.docx [file pcbi.1011802.s008.docx]

Dear Dr. Liu,

We are submitting our revised manuscript “Transcription factor interactions explain the context-dependent activity of CRX binding sites.” We revised the manuscript to address the comments of the reviewers. We have included several new analyses, new figure panels, new supplemental figures and new text.

We thank the reviewers for their constructive critiques and for their careful reading of the text. Their comments allowed us to strengthen the manuscript. We discuss our changes below, and we have attached a marked-up copy of the manuscript showing all changes. We note the following changes to the figures:

Fig 3: Panels have been re-labeled. Panel E is a new analysis of MPRA data.

Fig S2: Panels A, B, D, F, and G are new analyses responding to reviewer comments.

Fig S3: Panels C and E are new analyses.

Fig S4: This is a new figure presenting additional modeling.

Fig S5: This is a new figure presenting additional modeling.

Fig S6: This is a new figure showing the training history of the main figure models.

**Reviewer 1**

**1.1: “Can the authors speculate on why homotypic clusters could lead to silencer activity?”**

Response: Yes, there are a number of relevant papers with models or experimental results that suggest possible mechanisms by which homotypic clusters of binding sites could lead to silencing. We have added a section to the Discussion with new citations at the top of page 14.

**1.2: “Are there other examples of homotypic clusters leading to silencer activity, and are there examples of homotypic clusters leading to activation? Is this finding transcription factor specific, for example does it only apply to CRX, or certain types of transcription factors? Are there other examples of transcription factors that act as both activators and repressors where homotypic clusters are silencers or the opposite where such transcription factor clusters act as enhancers?”**

Response: Yes, homotypic clusters appear to play a variety of roles in *cis*-regulation, depending on the TF involved. We revised the Discussion to broaden our review of the existing literature, which includes cases where homotypic clusters appear to play a role in activation. There are a range of examples similar to what we observe with CRX, as well as contrasting examples. This revised text begins at the bottom of p. 13.

**1.3: “Are these findings cell type-specific? For example, within another cell context where CRX is expressed, do the authors think they’d find clusters of homotypic sites that would be activating or remain as silencers?”**

Response: We cannot provide a definitive answer to this question, however our results in *Crx^-/-^* retina indicate that *some* homotypic CRX site clusters are no longer repressive in other cellular contexts. These clusters are repressive when CRX is present and become activating when CRX is absent in *Crx^-/-^* retina. We *speculate* that this effect may mimic what might occur in photoreceptors vs bipolar cells: In photoreceptors, homotypic clusters are bound by CRX and are repressive. In bipolar cells, the same clusters may be bound by the CRX ortholog OTX2 and become activating. We address this in a revised Discussion on the middle of page 14.

**1.4: “• The introduction nicely talks about enhancer grammar, and mentions the billboard and enhanceosome models. The authors should also include the Transcription Factor Collective model (Junion Cell 2012) and the dependency grammar model (Jindal and Farley Developmental Cell 2021)”**

Response: We added the TF collective and the dependency grammar models to our discussion in the first paragraph of the Introduction. We included these two citations.

**1.5: “• In their second library, the authors find pairwise interactions between CRX and NEUROD1, and CRX and RORB contributed to stronger enhancer activity. Do the authors find examples of these combinatorial regulatory logics within known photoreceptor enhancers? Or can they find co-occurrences of these motifs within putative regulatory elements as marked by ATAC-seq or other epigenetic marks?”**

Response: All TFBSs included Library 2 co-occur with CRX sites in strong genomic enhancers much more often than they co-occur in weaker enhancers or silencers, suggesting that these TFBSs contribute to stronger elements in genomic sequences. This finding, which we described in a previous MPRA study (Friedman et al., 2021 PMID: 34486522), motivated the inclusion of these TFBSs in Library 2. The lab of co-author Joe Corbo also found that motifs for these TFs are enriched in accessible chromatin of rod photoreceptors (Hughes, et al., 2017, PMID: 28256534), which is additional support for the role of these TFs at native enhancers.

Thus these TFBSs were expected to contribute to stronger activity in the synthetic CREs. However, before the experiment reported in this manuscript, it was unclear whether these TFBSs contributed mainly via strong additive effects or by a positive interaction with CRX sites. Our model suggests that both types of effects contribute.

We have clarified these points in the section describing library 2 at the bottom of page 7.

**Reviewer 2**

**2.1: “Although the authors have listed their rational behind using MAVE-NN, there are already many different published tools to model and analyze MPRA data. Thus, it is important that the authors compare their measurements and results using few other tools for example, DEEP-STARR (PMID: 35551305) with details of the hyperparameters used to fit the models.”**

Response: While other published software tools are capable of modeling MPRA data, none of them are suited to our purpose, which is to learn interpretable parameters from synthetic CREs composed of TFBS building blocks. These tools are unsuitable for the following reasons:

1. Deep neural network models like DeepSTARR, DeepLiver (Gonzalez-Blas, et al. DOI: 10.1101/2022.12.08.519575), and MPRA-DragoNN (PMID: 31206543) take as input raw DNA sequence. They then learn the relevant binding sites in the raw sequence. Our synthetic CREs are composed of pre-defined binding sites. We use representations of binding sites, rather than raw sequence, as the input to our models.
2. The parameters of models like DeepSTARRther tools are not directly interpretable. These models require extensive interpretation pipelines to discover what the neural network learned about grammar. The parameters of MAVE-NN are interpretable as additive and interaction terms for TFBSs, which is what we seek to learn.
3. Our goal is *not* to predict the expression from raw sequence and then discover the relevant sequence features. Instead, we use MAVE-NN as a replacement framework for the prior statistical thermodynamic models that we and others previously used to model building-block synthetic CREs. (See for example Gertz 2009, PMID: 19029883; Zeigler 2014, PMID: 24288374; Fiore 2016, PMID: 27197208; King 2020, PMID: 32043966; Levo, 2015, PMID: 25762553; Sherman 2012, PMID: 22479169.)
4. Other modeling tools are designed for different tasks, such as accurately quantifying MPRA activity (MPRAnalyze, PMID: 31477158) or discriminating CREs from background genome sequence (MpraNet, PMID: 36350674). Models that do take TFBSs as input either do not include TFBS interaction terms (de Boer et al., 2020 PMID: 31792407), or are custom regression models (Ilias Georgakopoulos-Soares, et al., 2023 PMID: 37087538). As discussed in the original MAVE-NN paper (PMID: 35428271), MAVE-NN is to date the only non-custom, general purpose software package for training biophysically interpretable models on MPRA data. To our knowledge, no other published MPRA modeling tools exist that are suited for the analyses in this manuscript.

To address this concern, we extended our discussion of the rationale for using MAVE-NN and describe how MAVE-NN overcomes limitations of previous biophysical models that were trained on data from TFBS building-block libraries. This includes revised text in the third paragraph of the Introduction on page 3 and in the Results section beginning at the bottom of p. 4

**2.2: “One of the major limitations and what the authors have also noted in their manuscript is the lack of MPRA measurements without the basal Rho promoter which has multiple CRX sites. It would be best to perform this assay using a library without the Rho promoter and then model the effects both for the positive and negative interactions. This is not a big experiment and should have been done as new MPRA designs with synthetic CREs comprising of multiple different TFs were done for the results shown in Fig. 3.”**

Response: We tested Library 1 (previously published in White et al., 2016, PMID: 27783940) with both the Rho and the Hsp68 promoters. This allowed us to model the effect of the Rho promoter, which negatively interacts with CRX sites as shown in Fig. 2D. However, the purpose of Library 2 (with additional TFBSs) is different and does not require testing with an alternative promoter to support our claims. Library 2 was designed specifically to test whether other TFBSs interact with CRX sites, or whether they make independent contributions to activity. All CREs in Library 2 contain either *2 CRX sites, plus the Rho promoter sites*, or *3 CRX sites, plus the Rho promoter sites*. From this design, we infer that other TFBSs either do or do not interact with CRX sites, and that they overcome the negative homotypic interactions either via independent additive effects or heterotypic interactions. We believe that testing this Library 2 with the Hsp68 promoter will add no useful information. We discuss these points beginning at the bottom of page 7. See also response to 2.3 below.

**2.3: “It was also not clear which experiments and to what extent the MPRA data using Hsp68 promoter data was used. Comparison of results from both these results will also allow the authors to dissect the effects of the basal promoter used in their study. What is the correlation of MPRA activity from the two basal promoters i.e. Rho and Hsp68 and are the results consistent for example with the measured activity in terms of number of CRX or NRL sites?”**

Response: Our analysis already includes the effect of switching to the Rho promoter for library 1. For library 1 assayed in wild-type retina, the model was simultaneously trained on both Rho and Hsp68 promoter data. The Hsp68 promoter was taken as the baseline level in the model, allowing us to learn parameters that describe the effect of switching to the Rho promoter (see the Rho promoter column in Fig. 2 D).

In this manuscript, we are reporting a new model of our previously published data. In our earlier paper we extensively compared the activities of the Rho and Hsp68 versions of the library. We have thus not reproduced that analysis in this manuscript. Plots showing the differences between the Rho and Hsp68 libraries are included in Fig. 3 of the original publication (White, et al. 2016, PMID: 27783940). The two libraries are not strongly correlated: the negative interactions between multiple CRX sites in CREs with sites in the Rho promoter cause repression, something that does not occur with Hsp68.

To address this we revised our discussion of the Rho vs Hsp68 promoters in our explanation of the model in Fig. 2 on page 5 and the top of p. 6, and in the *Crx^-/-^* section on p. 7.

**2.4: “How do the authors explain their results in terms of biological context? Lot of different lineage-specific TF motifs were included in the synthetic CREs and measured but it would be good to know their function in vivo. For example, lot of these effects can be analyzed and measured in retinal pigmented epithelial (RPE) cell line. One way would be to look at the publicly available RPE cell line data such as ATAC-seq or if appropriate ChIP-seq data is available and can correlate the *cis*-regulatory grammar around the active and inactive genes. If such a grammar exists, then other way would be to use CRISPR to measure the effects CREs on gene activation or repression. This for sure would greatly improve the strength and outreach of this work.”**

Response: The TFs whose binding sites we included in synthetic CREs are known to play major roles in photoreceptor development, thus much about their *in vivo* function is known. Most of these TFs generally do not have roles in the RPE, which is not a relevant cell line for our study. We are modeling the CRX-mediated *cis-*regulatory grammar in photoreceptors. All data in this manuscript was collected from *ex vivo* retinas at P8, and thus our experimental system captures the relevant biological context for CRX. Our prior work has shown that electroporations of MPRA libraries primarily reach rod photoreceptors, which is our target cell type (Kwasnieski, et al., PMID: 23129659).

The distribution of the TFBS in these libraries near genes in different retinal cell types has previously been studied by the lab of co-author Joe Corbo, who published ATAC-seq data for photoreceptors (PMID: 30158147) and bipolar cells (PMID: 31633482). On the basis of this study, we previously examined the role of these TFBSs in native genomic CREs in an MPRA study (Friedman et al, PMID: 34486522). The synthetic CREs of Library 2 were designed based on this prior work looking at the *in vivo* function of these TFs.

To clarify these issues, we have expanded our discussion of the functional role of these TFs on page at the bottom of page 7. We also explain the selection of these TFs in the Discussion at the bottom of p. 12.

**Reviewer 3**

**3.1 There are discrepancies between the MAVE-NN models fit to dataset 1 (NFL and CRX binding sites only) and dataset 2 (CRX, NFL, NeuroD1, RORB and MAZ).**

**3.1A While the “dataset 1” model parameters support the “underlying hypothesis”, showing negative interaction terms for homotypic interactions between CRX and NFL and positive terms for heterotypic binding sites (Fig. 2D), the “dataset 2” model (Fig. 3D) loses the negative homotypic interaction for NFL, and a positive heterotypic interaction between 1 pair of TFs (CRX and RORB) binding sites is observed (interactions between CRX and the other three TFs are close to zero – no interaction, including for NFL, in contrast to the dataset 1 model – and the Crx-/- model in Fig. 2E). Thus, the simplified models, presented in Fig. 4A and 4B, are poorly supported by the “dataset 2” model.**

Response: We believe that dataset 1 and dataset 2 models both support our hypothesized mechanisms of CRX context dependence. The two models illustrate several ways of overcoming the strong effects of negative homotypic repression between CRX sites: (1) via positive heterotypic interactions (illustrated in Fig. 4A) and (2) via independent contributions of other TFBSs (illustrated in Fig. 4B).

The first mechanism (4A) is illustrated most clearly in library 1, which systematically tests all combinations of CRX and NRL in CREs composed of 1 to 4 binding sites. The stepwise increase in TFBS copy number makes this library well-powered to detect negative homotypic interactions and the positive interaction between CRX and NRL.

Library 2 primarily illustrates mechanism 2 (Fig. 4B), though negative homotypic and positive heterotypic interactions are also evident. The design of library 2 was motivated by the finding of our 2021 paper (Friedman, et al. *eLife* 2021, PMID: 34486522). We observed that genomic CRX-bound enhancers have a greater diversity of TFBSs than silencers. Library 2 was designed to test how TFBS diversity overcomes the negative effects of multiple CRX sites.

Because more TFBSs are included in library 2, we could not test all possible combinations of these TFBSs. To select informative sequences, we imposed constraints. The significant design differences in library 2 are: (1) all CREs are of constant length (5 sites), (2) TFBSs were only tested in one orientation, (3) every CRE included sites for three different TFs and included one central CRX site, and (4) TFBS copy number was not extensively varied. Additionally, the NRL sites in library 2 were taken from different genomic examples, and thus differed from the perfect NRL consensus sequence used in library 1.

The dataset 2 model indicates that cooperative interactions *with CRX* are not common among the TFBSs in this library. RORB sites tend to interact positively with CRX sites (Figs. 3D and E). The strongest positive interactions in the library are with NRL, but only when the NRL site is immediately 5’ of a CRX site (Fig. 3D, in the four outlier points on the box for high affinity NRL and in Fig. S3F). We conclude that (a) not all TFBSs contribute to activity via interactions with CRX, and (b) NRL sites that do not match the consensus sequence cooperatively interact with CRX only when adjacent.

We further address these points below in responses 3.1B and C. We revised the manuscript, as follows:

- We extended our discussion of the design and purpose of library 2 beginning at the bottom of p. 7.
- We marked the heatmap of the model (**Fig. S3F**) to highlight the strong positive heterotypic NRL-CRX interactions (noted by dashed boxes on the heat map.)
- We revised our discussion of Fig. 4 on p. beginning at the top of p. 11.

**3.1B The disagreement between NFL-NFL pairwise interactions in the MAVE-NN model for dataset 1 versus 2 could be due to (1) differences in the MPRA reporter design and/or (2) overfitting of the data by the MAVE-NN models. Regardless, the generalizability of the underlying hypothesis based on the data and models in the study remains unclear. (If the NFL-NFL homotypic and heterotypic NFL-CRX interactions change so much between dataset 1 and dataset 2 models, how confident can we be in the other homotypic and heterotypic CRX interactions?) It would be instructive to visualize “dataset 2”, as was done for “dataset 1” in Fig. 1B, examining each TF separately, to see the synergistic impacts of the other TFs on enhancer activity of sequences containing the CRX sites. This will help determine whether the issue is (1) differences in reporter architecture and context or (2) a limitation of the MAVE-NN model.**

Response: For reasons partially discussed under 3.1A, we don’t think the models are inconsistent, and we believe that the main differences are due to reporter library design. The differences in NRL interactions are likely due to the fact that we did not systematically increase NRL copy number beyond 2, that the orientation and position of one CRX site was kept constant (at the CRE center), and that high and medium affinity NRL site sequences differed from the consensus sequence in library 1.

In response to this comment, we added a new figure panel, Fig 3E, analogous to Fig 1B. All CREs in library 2 have sites for CRX plus two other TFs, thus it is not possible to show individual TF effects as in Fig 1B. However, this new figure reveals three key patterns that support our hypothesis: (1) CREs with RORB sites have the highest activity, supporting an interaction with CRX. (2) CREs with three CRX sites express below levels of CREs with two CRX sites, showing a quantitative effect of negative homotypic interactions. (3) Most CREs are active above basal, which supports the claim that the other TFBS overcome the impact of negative homotypic interactions.

We revised our discussion of Figure 3 and dataset 2 throughout that section to address the above concerns. See section beginning at the bottom of p. 8.

**3.1C Thus, there is a need to find additional support for the simple models put forth in Fig. 4A and Fig 4B. There are several MPRA datasets and models that could examine the hypothesis further, specifically looking at CRX or CRX TF family members (comparing whether the number of homotypic binding sites shows a similar impact on enhancer activity and how heterotypic binding sites change this relationship). (See publications of Carl de Boer, data and existing models (e.g., in yeast, 2020 Nature), also CAGI challenge data and models). Of course, generalizability of the simple models to endogenous contexts would increase the impact of the work further, and that could be accomplished through motif analysis of nascent transcription data…**

Response: To support our hypothesized mechanisms, we trained MAVE-NN models on two additional published MPRA datasets from Smith, et. al. 2013 (PMID: 23892608). These new models are presented in Supplementary Figs. 4 and 5, discussed on page 9.

We modeled data from a library of synthetic CREs composed of 12 liver-specific TFs, tested in HepG2 cells and in mouse liver *in vivo*. These models indicate that (a) negative homotypic interactions are a feature of other cell-type specific TFs and (b) additive, independent effects overcome negative homotypic interactions, even in the absence of strong positive cooperativity (the model in Fig. 4B).

In addition to the new models of other MPRA datasets, we provide additional support for our models by citing multiple examples of negative homotypic effects in other systems.

To support the biological relevance of our hypothesis for CRX, we extended the discussion of our prior work, which addresses the above comment. Specifically, we previously showed:

1. In genomic CREs, homotypic clusters of CRX binding sites are repressive (White 2013, White 2016, Friedman 2021). In White 2016, we showed the effects of homotypic clusters in synthetic CREs in dataset 1 (Fig. 1B of this manuscript) are recapitulated in genomic CREs (Figs 2A and 3A in White 2016).
2. CRX-bound enhancers are enriched for heterotypic binding sites, compared to silencers (Fig. 3 in Friedman 2021).

Thus, our hypothesis in this paper was not developed only from dataset 1, but rather from multiple MPRA studies with both genomic and synthetic CREs.

What was missing in the prior work was an explanatory mechanism - it was unclear how binding sites CRX could have both repressive and activating effects in different contexts. Our models here suggest a mechanism, presented in Figs 4A and 4B, that account for what we have extensively observed in genomic CREs.

We revised our discussion of supporting findings from our prior work with genomic enhancers at the bottom of p. 12

**3.2 If the MAVE-NN model of dataset 2 is robust and accurate, then the interactions describing homotypic and heterotypic interactions are more complex than presented in Fig. 4.**

**3.2A From Fig. S3C, we see that the interaction term between high-affinity NRL and high-affinity CRX depends on the spacing between the binding sites, as there is a positive interaction when the binding sites are adjacent but not when there’s a greater spacing. Several of the other heterotypic interactions also show spacing-dependent interactions.**

Response: We agree that TFBS interactions are more complex than what is presented in Fig. 4. The purpose of the simplified models of Fig. 4 is *not* to capture all of those effects, but rather to illustrate the novel hypothesis in this paper: that context-dependent activation and repression can achieved by a balance among negative homotypic interactions, independent ‘additive’ effects, and positive heterotypic cooperativity. This new hypothesis explains how a transcriptional activator (like CRX) may also, in a different context, act repressively at silencers. We believe that Figs. 4A and 4B are an accurate summary of key effects in the models of datasets 1 and 2. But these simple models are not meant to account for the full complexity of TF interactions.

We revised the text to emphasize that Fig. 4 is meant to illustrate the proposed novel mechanism but is not meant to capture or deny the full complexity of the effects that determine CRE activity. We acknowledge the limitation of the simple model of Fig 4 in the following places: the top of p. 9, bottom of p. 10, and the last paragraph of the discussion on p. 14,

**3.2B The results for moderate affinity binding sites are often different from the high affinity RFX result, adding another layer of complexity. What is the biological interpretation?**

Response: Because the parameters of the medium affinity sites are usually weaker than those for high affinity parameters, we assume that this is largely an effect of reduced occupancy. However, we can’t rule out that related TFs with similar specificities may compete for binding at medium affinity sites, producing a qualitative difference in effect. We note this interpretation at the top of p. 9.

**3.3 Loell et al. need to demonstrate that the MAVE-NN models are robustly fit. There is no justification for the parameters selected for the MAVE-NN models nor are the results shown to be robust to the parameter choices.”**

Response: We made the following revisions to show the robustness of our models:

1. We generated bootstrap estimates of parameter uncertainties. These are shown in new figure panels S2B and G, and S3E. See p. 5 of the text.
2. We note in Methods (p. 15) that hyperparameters were initially selected based on the MAVE-NN defaults. We made minor adjustments guided by the best fit to the validation set.
3. We present the model training histories in a new figure S6, which is referenced in Methods (p. 15).

**3.4 The design of the dataset 2 MPRA is circular. NRL, NEUROD1, RORB and MAZ motifs were selected from sites identified in genomic strong enhancers that lose activity when the corresponding motif is deleted. The hypothesis from Fig. 1B is that binding of these other TFs can overcome repression of multiple CRX binding sites... but, given the selection criteria for the other TFs, this impact might be independent of CRX binding sites.**

Response: Our description of the design of dataset 2 was unclear. We picked NRL, NEUROD1, RORB and MAZ because we previously found that these motifs were enriched in CRX-bound genomic enhancers, relative to silencers (Friedman 2021, PMID: 34486522). We then needed to pick one high and one medium affinity version of each binding site to construct the synthetic CREs. Rather than select the consensus sequence and an arbitrary weaker PWM match, we picked high and medium affinity motif matches from a previous mutagenesis experiment, showing that those specific sequences are functional in at least one context. Motif sequences were chosen if mutations caused *some* loss of activity, which we did not explain clearly. This loss may or may not be independent of CRX sites.

We revised our explanation of the purpose and design of library 2 at the bottom of p. 7 and in the Methods on page 15.

**3.5 It would be beneficial to explain where the distribution of interactions from Fig. 3E came from – is it a distribution across positions?**

Response: Yes, this is a distribution across positions. We have revised the previously unclear text to discuss this figure (p. 8), which is now **Fig. 3D**.

**3.6 For comparison of MAVE-NN models (Fig. 2A, Fig. S2A), additional metrics of model prediction should be included in the comparison (e.g., Pearson correlation of prediction).**

Response: We added Pearson correlation plots as Fig. S2 A and F and S3 C. The results do not change the rank order of the models, though the difference in performance are greater when measured by predictive information.

**3.7 For the “dataset 1” pairwise model, there’s a line of strong positive (or negative) interactions between moderate affinity reverse-strand CRX motif (m) and all other factors in adjacent positions (both Fig. S2D and E). What is the biological explanation for that or is there a problem with the way the model is explaining variance?”**

Response: The reverse medium affinity CRX motif is indeed an outlier. We tested the robustness of the model parameters for this result and found that the reverse site was consistently different from the forward site in multiple independently trained models, initiated from different random seeds. We added a new figure panel (**S2D**) to show this. The parameters reflect a real trend in the data: A single reverse medium site has about 5 times lower MPRA activity than the forward version, indicating a weaker additive effect. The reverse site also exhibits different interactions. For example, CREs with 4 reverse sites show a much weaker negative homotypic effect.

Our speculative biological explanation is that one of the other homeodomain TFs expressed photoreceptors binds competitively to these sites, and that its effect is strong when bound 3’ of CRX or NRL. The partial persistence of this effect in Crx -/- retina suggests that there is another factor able to interact with both CRX and OTX2, which are close homologs. We now discuss this outlier site in the middle of p. 6 where we reference Fig S2D.
